# Advanced Therapy Medicinal Products for Eye Diseases: Goals and Challenges

**DOI:** 10.3390/pharmaceutics13111819

**Published:** 2021-11-01

**Authors:** Yolanda Diebold, Laura García-Posadas

**Affiliations:** 1 Ocular Surface Group, Instituto de Oftalmobiología Aplicada (IOBA), Universidad de Valladolid, 47011 Valladolid, Spain; lgarciap@ioba.med.uva.es; 2 Centro de Investigación Biomédica en Red de Bioingeniería, Biomateriales y Nanomedicina (CIBER-BBN), Instituto de Salud Carlos III, 28029 Madrid, Spain

Advanced therapy medicinal products (ATMPs) are a novel class of medicines with enormous potential to improve treatments for a wide range of diseases, including those affecting eye structures. The purpose of ATMPs is to replace or regenerate human cells, tissues, or even organs to restore their normal functions. These complex biological products mainly include gene therapies, somatic cell-based therapies, and tissue-engineered products; the last two are frequently referred to as regenerative medicine. The development of ATMPs is scientifically challenging, but assessment of their clinical success in actual patients is even a more difficult goal to achieve. This fact partly accounts for the low numbers of ATMPs in the market.

The eye is a suitable organ to apply ATMPs for different reasons such as small dimensions, compartmentalized anatomical structure, and good accessibility for treatments, among others; however, there are few examples of ATMPs specific for ocular diseases. Eye structures pose specific challenges which make it difficult for basic and clinical scientists to develop this kind of complex medicines and successfully reach the clinical trial phase.

The aim of this Special Issue is to provide an overview of the current progress in the application of ATMPs to restore functionality in diseased ocular structures. An additional aim is to update the challenges that this novel class of medicines must deal with to effectively reach patients. A series of four research and six review articles is brought together to provide updated information about some of those challenges. Authors who kindly contributed are well-known experts in pre-clinical or clinical research involving the development of ATMPs for the eye, and some of them pioneered their application for specific ocular diseases.

López-Paniagua M. et al. [1] analyze the characteristics that make the eye an ideal organ to receive ATMPs. In their comprehensive review paper, these authors identify all groups of ATMPs according to the European Medicines Agency and deal with the legal framework that regulates their development and classification in the European Union. Regulatory principles associated with this type of innovative medicinal products are revised using specific examples for the treatment of eye diseases.

Cell-based therapy for eye diseases is one of the most successful examples of current regenerative medicine. Two excellent review papers deal with this aspect of ATMPs, one of them focused on cell therapy for diseases leading to corneal blindness [2] and the other one on cell replacement for retinal or optic nerve degeneration [3]. Calonge M. et al. [2] present the goals and challenges of stem cell-based therapy to treat corneal blindness caused by limbal stem cell deficiency, which affects the stem cell niche located at the corneal limbus. This review paper revises the challenges related to the development of cell-based products for the anterior part of the eye and the importance of the close interaction between basic and clinical scientists to succeed. On the other hand, Coco-Martin R. et al. [3] focus their review in the posterior part of the eye, specifically in the potential of cell therapies to restore or replace damaged and/or lost cells in retinal degenerative and optic nerve diseases. The authors describe the available cell sources and the challenges involved in the development of such treatments. They also present those issues that remain to be solved concerning the clinical translation of cell-based therapy for the retina, such as specific retinal subtypes enrichment, cell survival and cell delivery to the target site, and the evaluation of the risk of tumor formation caused by transplanted cells.

The conjunctiva is an underestimated yet incredibly important tissue in maintaining ocular surface homeostasis and contributes to eye protection. Diebold Y. and García-Posadas L. [4] evidence in their review paper how the conjunctiva would benefit from ATMP development because of the relevant pathology that impairs the ocular surface by affecting this tissue. They also provide readers with promising pre-clinical examples mostly oriented to cell therapy and tissue engineering.

Biomaterials are key elements to consider for tissue-engineered products because of their tunable properties. They are used to prepare scaffolds suitable to support cells in 3D tissue equivalents, to deliver active agents, or even to provide tissues with beneficial properties by themselves. Hydrogels are currently being investigated with great interest for ocular tissue-engineering applications. In the eye, the cornea is a good candidate to explore the biocompatibility, mechanical properties, and chemical structure of hydrogels for corneal replacement in different pathologies. Bhattacharjee and Ahearne [5] explore in their review paper different aspects of hydrogel crosslinking for corneal tissue engineering and repair. Issues such as crosslinking techniques, crosslinking chemical additives, and factors influencing crosslinking success including immunogenicity and toxicity are discussed, as well as the limitations and prospects of crosslinking strategies in this field. The research article by Nagai N. et al. [6] present a novel biopolymer (MPCP) with hydrating properties that helps to normalize the tear film in an animal model of the severe immune-based, inflammatory disorder that impairs the homeostasis of the ocular surface known as dry eye disease. Using a specific polymerization procedure, MPCP polymer enhanced tear film volume and prolonged the tear film breakup time in disease animal, indicating promising therapeutic properties.

Another example of advanced therapy is the 3D bioprinting of eye tissues that allows the construction of scaffolds from different biomaterials in which appropriate cell types are incorporated in a custom-fabricated way. Ruiz-Alonso S. et al. [7] update the application of this revolutionary technology to the ocular tissue regeneration field, paying special attention to the manufacture of relevant ocular tissues such as cornea, retina, and conjunctiva in preclinical studies. Issues related to current bioprinting methods and ethical and regulatory aspects are also analyzed.

Other experimental approaches to the design and potential application of biopolymeric scaffolds for ocular tissue regeneration in the context of diverse pathologies are explored in a series of three original research articles. One first approach is the fabrication of fiber scaffolds using the electrospinning technique for conjunctiva regeneration. Bosworth L. A. et al. [8] explored the potential of electrospun scaffolds composed from poly (ε-caprolactone) and decellularized tissue matrices, such as small intestinal submucosa or urinary bladder, to culture human conjunctival epithelial cells and induce their stratification at the air/liquid interface. The bioactive scaffolds created induced an in vitro cell response in terms of changes in cell morphology and stratification. Islam M. M. et al. [9] proposed a double-crosslinking manufacturing approach to fabricate collagen scaffolds with improved mechanical and functional properties, as well as cell biocompatibility. This novel approach would facilitate the use of collagen-based implants in corneal regeneration. Finally, Sánchez-Porras D. et al. [10] evaluated four different protocols to decellularize porcine limbus and further recellularize them with a limbal epithelial cell line and human adipose-derived mesenchymal stem cells. Limbal substitutes generated maintained transparency and other specific limbal characteristics, preliminarily indicating a potential for their use in limbal tissue regeneration.

We would like to thank all the participants in this Special Issue (authors, reviewers, and assistant editors, especially Mr. Albert Yang) for their rigorous work that allowed us to provide the scientific community with an updated collection of research papers and reviews about ATMPs for eye diseases. We hope the readers find their contents useful.
